# Prevalence of cardiovascular diseases and associated factors among adults from southwest Iran: Baseline data from Hoveyzeh Cohort Study

**DOI:** 10.1186/s12872-022-02746-y

**Published:** 2022-07-08

**Authors:** Nader Saki, Majid Karandish, Bahman Cheraghian, Habib Heybar, Seyed Jalal Hashemi, Maryam Azhdari

**Affiliations:** 1 Hearing Research Center, Clinical Sciences Research Institute, Ahvaz Jundishapur University of Medical Sciences, Ahvaz, Iran; 2 Nutrition and Metabolic Diseases Research Center, Clinical Sciences Research Institute, Ahvaz Jundishapur University of Medical Sciences, Ahvaz, Iran; 3 Department of Biostatistics and Epidemiology, School of Public Health, Alimentary Tract Research Center, Clinical Sciences Research Institute, Ahvaz Jundishapur University of Medical Sciences, Ahvaz, Iran; 4 Atherosclerosis Research Center, Ahvaz Jundishapur University of Medical Sciences, Ahvaz, Iran; 5 Department of Internal Medicine, School of Medicine, Alimentary Tract Research Center, Ahvaz Jundishapur University of Medical Sciences, Ahvaz, Iran; 6 Department of Nutrition, School of Public Health, Shahid Sadoughi University of Medical Sciences and Health Services, Yazd, Iran

**Keywords:** Cardiovascular diseases (CVDs), Anthropometric measurements, Prevalence, Risk factors, Lipid profiles, Hypertension

## Abstract

**Background:**

Cardiovascular diseases (CVDs) were the number one cause of death in Iran. The main risk factors of CVDs include unhealthy lifestyles, insulin resistance, hypertension (HTN), and hyperlipidemia. Given that there are modifiable risk factors for CVDs, this cross-sectional study aimed to evaluate the prevalence of CVDs and their risk factors among adults.

**Methods:**

The present cross-sectional study was conducted on 9828 adults 35–70 years (both sexes). The demographic data, lifestyle habits, anthropometric data, and clinical and biochemical parameters were collected from the baseline data of the Hoveyzeh Cohort Study. The odds ratio (OR) of CVDs was assessed by multivariable logistic regression.

**Results:**

The prevalence of CVDs was higher in females than males (16.2 vs. 12.6, *p* ≤ 0.001). The prevalence of CVDs was related to age, gender, marital status, lifestyle, anthropometric measurements, cholesterol, high-density lipoprotein, HTN, and fasting plasma glucose (FPG) (*p* ≤ 0.05). The participants aged 65–70 y showed the highest odds of CVDs (OR: 3.97, 95% CI: (3.14, 5.01), (*p* ≤ 0.001)). Males (OR: 1.76, 95% CI: (1.51, 2.05), *p* ≤ 0.001), married status (OR: 1.63, 95% CI: (1.08, 2.47), *p* = 0.021), more using a mobile phone (OR: 1.26, 95% CI: (1.09, 1.46), *p* ≤ 0.002), and smoking cigarettes (OR: 1.44, 95% CI: (1.24, 1.68), *p* ≤ 0.001) associated with CVDs. Higher odds of CVDs were related to low physical activity (PA) (OR: 1.56, 95% CI: (1.34, 1.8), *p* ≤ 0.001), body mass index > 30 (OR: 1.68, 95% CI: (1.01, 2.8), *p* ≤ 0.047). Moreover, odds of CVDs were related to systolic blood pressure (SBP) ≥ 140 mm Hg (OR: 1.25, 95% CI: (1.04, 1.51), *p* = 0.017), FPG = 100–126 mg/dl (OR: 1.24, 95% CI: (1.07, 1.43), *p* = 0.003), and FPG > 126 mg/dl (OR: 1.71, 95% CI: (1.47, 1. 98), *p* ≤ 0.001).

**Conclusion:**

The present study showed the main risk factors of CVDs were older age, married status, using a mobile phone, low PA, smoking, obesity, and abnormal FPG and SBP. The lower odds of CVDs were found in the participants with normal cholesterol.

**Supplementary Information:**

The online version contains supplementary material available at 10.1186/s12872-022-02746-y.

## Introduction

Cardiovascular diseases (CVDs) are a group of disorders of the heart and blood vessels, including coronary heart disease, cerebrovascular disease, rheumatic heart disease, and other conditions. They are the leading cause of death globally [1]. Their Symptoms may be caused anywhere in the body and vary depending on the specific situation. According to the World Health Organization (WHO), an estimated 17.9 million lives are taken by CVDs each year. More than 80% of CVD deaths are due to heart attacks and strokes, and one-third of these deaths occur prematurely in people under 70 years of age [1, 2].

The first leading cause of mortality and a million disability-adjusted life years (DALYs) in Iran emanated from CVDs. Moreover, CVDs can lead to 46% of all deaths and 20%-23% of the burden of disease in Iran [3]. Compared to 2005, DALYs related to CVDs are predicted twofold in 2025 among Iranians aged ≥ 30 years. However, the prevalence among men will still be higher than among women; the difference will be less in 2025 [4].

The risk factors of CVDs are a variety. Some of them include inappropriate diet, physical inactivity, smoking, harmful use of alcohol, industrialization, urbanization, cultural changes, socioeconomic status, wealth index, and increasing life expectancy [1, 3, 5, 6]. In addition, people with high glucose, hypertension (HTN), hyperlipidemia, and increased body mass index (BMI) may be more exposed to the risk factors of CVDs [2, 5].

Although the evidence for the association between some risk factors and CVDs is relatively strong, clarifying the high-risk patients, the trend of prevalence and risk factors of CVDs seem necessary based on the demographic characteristics, history of diseases, socioeconomic status, and lifestyle behaviors in the different populations [1, 5, 7]. On the other hand, as was previously published, some non-communicable diseases (obesity, prediabetes, dyslipidemia, and HTN) were high among adults from Hoveyzeh [8]. So, the present study was designed to determine the prevalence and risk factors of CVDs among adults aged 35–70 years from southwest Iran (Baseline data from Hoveyzeh Cohort Study).

## Methods

### Study population and sampling methods

**Fig.1:**
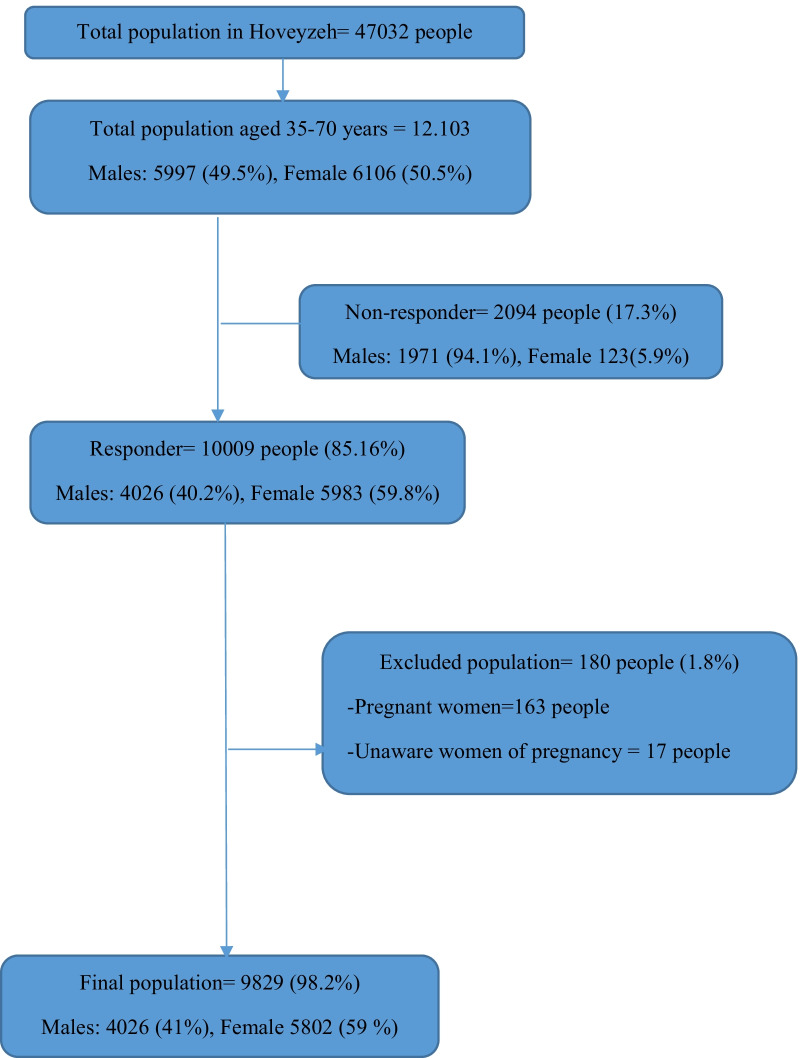
Flowchart of the participation in the present study (Hoveyzeh Cohort Study)

The present cross-sectional study was conducted on the baseline data from the Prospective Epidemiological Studies of the Iranian Adult and Neonates (PERSIAN), Hoveyzeh Cohort Study (HCS). ‘in the HCS, it was targeted to sample the entire study population (adults aged 35–70), but only 85.16% agreed to respond to the survey. However, the total adults aged 35–70 years was 12,103 in Hoveyzeh (Arab community), Khuzestan Province, southwest Iran,. Therefore, 10,009 people (85.16%) were recruited in HCS [8]. Data collection was from May 23, 2016, to August 28, 2018. The Non-responder of the study were 2094 people (Responders = 10,009) (the causes were mentioned in Fig. 1). The pregnant women (*n* = 163) and women who were unaware of pregnancy (*n* = 17) were excluded from the study *N* = 180 excluded population (Fig. 1). Therefore, 9829of 10,009 had the criteria for the present study. The pregnant women (*n* = 163) and women who were unaware of pregnancy (*n* = 17) were excluded from the survey *N* = 180 excluded population (Fig. 1).

A voluntary and written informed consent was obtained from the research participants. The details of their recruitments were previously published [8]. The Ethics Committee of Ahvaz Jundishapur University of Medical Sciences has approved the present study (Ethical code: IR.AJUMS.REC.1398.761).

### Measurements

The following information was obtained from all participants:

- Demographic data (sex, age, marital status) in the past year and Wealth index in the past and current.
- Physical activity (PA) in the past year and personal habits (using a cell phone (mobile phone), drinking alcohol habit, smoking status, and drug use) in the past and current
- History of diseases (cardiac ischemic (CI), myocardial infarction (MI), stroke, HTN, prediabetes, and type 2 diabetes mellitus (T2DM)).
- Anthropometric measurements (BMI, waist circumference (WC), high waist-to-hip ratio (WHR), and hip circumferences (HC))
- Clinical measures (systolic blood pressure (SBP), and diastolic blood pressure (DBP)), biochemical parameters (fasting plasma glucose (FPG), triglyceride (TG), total cholesterol (TC), and high-density lipoprotein cholesterol (HDL-C)).

Some information was gathered through interviewer-administered questionnaires by the trained interviewers. CVDs were defined as stroke, CI, and MI in the present study.

The self-reported daily physical activity questionnaire was used to measure metabolic equivalent rates (METs) for all participants' activities [9] (Additional file 1: Table S1). It was categorized into three groups (quantile): the less quantile (inactive), the moderate quantile (moderately active), and the most quantile (active). The questionnaire details are available in (Additional file 1: Table S.1).

The international wealth index was used to measure the socio-economic status of households and calculated by a principal component analysis (PCA), including the means of information on households’ possession (freezers, TV, motorbike, cell phone, car, vacuum cleaners, access to the internet, washing machines, and computers) and household utilities (house ownership and the number of rooms per capita). The wealth index score was categorized into five groups: poorest, poor, moderate, rich, and richest [10].

Anthropometric data were measured in a fasting state in the morning. Height (cm) was measured using a stadiometer (Seca 206), weight (kg) was measured using a standing scale (Seca 755), and WC and HC (cm) were measured using Seca locked tape meters. The WHR and BMI (kg/m^2^) were calculated by dividing WC (cm) by HC (cm) and body weight (kg) by the square of the height (m^2^), respectively. BMI were categorized four groups: (a) underweight: BMI < 18.5; (b) normal range: BMI = 18.5 to 24.9, (c) overweight: BMI = 25.0–29.9; obese: BMI > 30. Abnormal WC was defined as ≥ 102 cm in men and ≥ 88 cm in women. A healthy WHR was considered as ≤ 0.85 and ≤ 0.90 for women and men, respectively.

The measures of DBP and SBP were conducted twice (10 min intervals) on each arm, following standard guidelines by Riester Sphygmomanometers. HTN was considered to have an SBP ≥ 140 mm Hg and a DBP ≥ 90 mm Hg at the baseline, have a self-reported history of physician-diagnosed HTN or take antihypertensive medication [11].

The trained laboratory staff collected blood samples at 10–12 h of fasting. First, the serums were obtained by centrifuging the blood samples for 10 min at 3000 rpm at room temperature (Sigma, Germany). Then, the autoanalyzer (BT 1500, Biotecnica Instruments, Italy) was used to measure the required serum levels.

### Statistical analysis

The analysis of continuous and categorical variables was conducted by means ± standard deviations (SD) and frequency (number (%)), respectively. In addition, the Chi-square test was used to compare the sociodemographic and lifestyle characteristics by gender. Further, the Chi-square test examined the association of sociodemographic and lifestyle factors with CVD. Finally, the multivariable logistic regression analysis explored all studied risk factors for CVD. The potential cofounders have included sociodemographic and lifestyle characteristics (age, gender, wealth score, marital status, using a mobile phone, smoking cigarettes, drinking alcohol, PA, anthropometric measurements (BMI, WC, WHR), and lipid profiles (TG, cholesterol, HDL)). The backward approach (starting with a full model and dropping the variables) was used for a variable selection. Parameters of the model were estimated through the maximum likelihood. The unadjusted analyses were performed for the potential confounders, and the variables with *p* < 0.25 in the unadjusted analyses were considered as confounders and included in the final model. The statistical analyses were conducted using the SPSS statistical software package, version 16.0 (SPSS, Inc, Chicago, Illinois, USA). *P* < 0.05 was considered statistically significant using 2-tailed tests.

## Results

### Gender-specific sociodemographic and lifestyle characteristics of the respondents

An overall of 9829 participants was included in the present study, of whom 59% were female, and 41% were male. All sociodemographic and lifestyle characteristics of the study population except MI (*p* = 0.056) and stroke ((*p* = 0.39) were shown a significant difference between the sexes (Table 1). Only 7.2% of the study population was 65–70 years. The prevalence of history of HTN (26.5%), CI (13.6%), prediabetes (23%), and diabetes (17%) was common in Hoveyzeh.

**Table 1:** Gender-specific Sociodemographic and lifestyle characteristics of the study population

Characteristics	Overall ((*N*) %)	Male ((*N*) %)	Female ((*N*) %)	*P*-value
*Age*				
35–45	3836 (39)	1526 (37.9)	2310 (39.8)	0.018
45–55	3234 (32.9)	1312 (32.6)	1922 (33.1)	
55–65	2052 (20.9)	902 (22.4)	1150 (19.8)	
65–70	706 (7.2)	286 (7.1)	420 (7.2)	
*Marital status*				
Single	343 (3.5)	40 (1)	303 (5.2)	≤ 0.001
Married	8603 (87.5)	3949 (98.1)	4654 (80.2)	
Widow	717 (7.3)	19 (0.5)	698 (12)	
divorced	165 (1.7)	18 (0.4)	147 (2.5)	
*Wealth index*				
Poorest	1959 (19.9)	647 (16.1)	1312 (22.6)	≤ 0.001
Poor	1999 (20.3)	729 (18.1)	1270 (21.9)	
Moderate	1952 (19.9)	821(20.4)	1131(19.5)	
Rich	1979 (20.1)	880 (21.9)	1099 (18.9)	
Richest	1939 (19.7)	949 (23.6)	990 (17.1)	
*Having*				
Hypertension	2608 (26.5)	943 (23.4)	1665 (28.7)	≤ 0.001
Cardiac Ischemic	1340 (13.6)	447(11.1)	893 (15.4)	≤ 0.001
Myocardial infarction	184 (1.9)	88 (2.2)	96 (1.7)	0.056
Stroke	158 (1.6)	70 (1.7)	88 (1.5)	0.39
Prediabetes *	2265 (23)	842 (37.2)	1423 (62.8)	≤ 0.001
Diabetes *	1691 (17)	726 (42.9)	965 (57.1)	≤ 0.001
Using mobile	7598 (77.3)	3794 (94.2)	3804 (95.6)	≤ 0.001
Smoking cigarette	2075 (21.1)	1635 (40.6)	440 (7.6)	≤ 0.001
Drinking Alcohol	197 (2)	191 (4.7)	6 (0.1)	≤ 0.001
*Physical activity*				
Quantile 1 (Inactive)	3278 (33.4)	1547 (38.4)	1731 (29.8)	≤ 0.001
Quantile 2 (Moderate)	3283 (33.4)	840 (20.9)	2443 (42.1)	
Quantile 3 (Active)	3267 (33.2)	1639 (40.7)	1628 (28.1)	
*Body mass index (kg/cm*^*2*^*)*				
< 18.5	146 (1.5)	70 (1.7)	76 (1.3)	≤ 0.001
18.5–24.99	2213 (22.5)	1146 (28.5)	1067 (18.4)	
25–29.99	3652 (37.2)	1706 (42.4)	1946 (33.5)	
> 30	3817 (38.8)	1104 (27.4)	2713 (46.8)	

The Chi-square test was used to assess the significant difference among sociodemographic and lifestyle characteristics by genders. *P*-value ≤ 0.05 was considered significant

*Based on Fasting Blood Sugar, it was categorized

The distribution of respondents was similar in terms of wealth scores and PA levels. Most of the surveyed population (76%) showed abnormal BMI (overweight (37.2%) and obesity (38.8%)).

The history of HTN, CI, prediabetes, and diabetes in females was significantly higher than in males (*p* ≤ 0.001). More mobile phone use was shown significantly in females than males (*p* ≤ 0.001). The history of smoking, drinking alcohol, and using drug abuse was significantly higher in males than females (*p* ≤ 0.001). Based on the PA levels, there was a significant difference between genders. However, most females (42.1%) had moderate levels of PA, and the percent of women with inactive and active PA levels were similar (29.8 and 28.1%, respectively). Only 20.9% of males had moderate PA levels, while 38.4 and 40.7% were inactive and active, respectively. The prevalence of overweight or obesity was significantly higher in females than in males (*p* ≤ 0.001).

### The prevalence of CVDs by the studied characteristics

Table 2 presents the prevalence of CVDs by the studied characteristics. The increasing trend in age showed a higher prevalence of CVDs (7.5% (35–45 y) vs. 27.9% (65–70 y), *p* ≤ 0.001). The prevalence of CVDs was higher in females than in males (16.5% vs. 12.6%, *p* ≤ 0.001). The most and less prevalence of CVDs were reported in widow (24.8%) and single (7.6%) participants (*p* ≤ 0.001).

**Table 2:** Prevalence of cardiovascular diseases (CVDs) ^◙^ by the studied characteristics

Characteristics	CVDs		*p*-value
	No	Yes	
	Number (%)	Number (%)	
*Age (years)*			
35- 45	3549 (92.5%)	287 (7.5%)	≤ 0.001
45–55	2750 (85.0%)	484 (15.0%)	
55–65	1558 (75.9%)	494 (24.1%)	
65–70	509 (72.1%)	197 (27.9%)	
*Gender*			
Male	3520 (87.4%)	506 (12.6%)	≤ 0.001
Female	4846 (83.5%)	956 (16.5%)	
*Marital status*			
Single	317 (92.4%)	26 (7.6%)	≤ 0.001
Married	7361 (85.6%)	1242 (14.4%)	
Widow	539 (75.2%)	178 (24.8%)	
divorced	149 (90.3%)	16 (9.7%)	
*Wealth index*			
Poorest	1656 (84.5%)	303 (15.5%)	0.22
Poor	1732 (86.6%)	267 (13.4%)	
Moderate	1667 (85.4%)	285 (14.6%)	
Rich	1678 (84.8%)	301 (15.2%)	
Richest	1633 (84.2%)	306 (15.8%)	
*Using a mobile*			
Yes	6485 (85.4%)	1113 (14.6%)	0.243
No	1881 (84.3%)	349 (15.7%)	
*Smoking cigarette*			
Yes	6675 (86.1%)	1078 (13.9%)	≤ 0.001
No	1691 (81.5%)	384 (18.5%)	
*Drinking alcohol*			
Yes	167 (84.8%)	30 (15.2%)	0.888
No	8199 (85.1%)	1432 (14.9%)	
*Physical activity*			
Quantile 1 (Inactive)	2598 (79.3%)	680 (20.7%)	≤ 0.001
Quantile 2 (Moderate)	2874 (86.9%)	434 (13.1%)	
Quantile 3 (Active)	2894 (89.3%)	348 (10.7%)	
*Body mass index (kg/cm*^*2*^*)*			
< 18.5	127 (87.0%)	19 (13.0%)	≤ 0.001
18.5–24.99	1939 (87.6%)	274 (12.4%)	
25–29.99	3150 (86.3%)	502 (13.7%)	
> 30	3150 (82.5%)	667 (17.5%)	
*Waist circumstance (WC)(cm)*			
Normal**	3047 (89.0%)	375 (11.0%)	≤ 0.001
Abnormal	5319 (83.0%)	1087 (17.0%)	
*Waist-to-hip ratio (WHR)*			
Normal**	893 (91.8%)	80 (8.2%)	≤ 0.001
Abnormal	7473 (84.4%)	1382 (15.6%)	
*Triglyceride (mg/dl)*			
< 150	4674 (84.9%)	830 (15.1%)	0.521
≥ 150	3692 (85.4%)	63 (14.6%)	
*Cholesterol (mg/dl)*			
< 200	5285 (84.3%)	983 (15.7%)	0.003
** ≥ **200	3081 (86.5%)	479 (13.5%)	
*High-density lipoprotein cholesterol (HDL-C) (mg/dl)*			
Normal *	5386 (86.1%)	872 (13.9%)	0.001
Abnormal	2980 (83.5%)	590 (16.5%)	
*Systolic blood pressure (mmHg)*			
< 140	613 (76.9%)	1278 (14.2%)	≤ 0.001
≥ 140	7753 (85.8%)	184 (23.1%)	
*Diastolic blood pressure (mmHg)*			
< 90	7853 (85.4%)	1341 (14.6%)	0.002
≥ 90	513 (80.9%)	121 (19.1%)	
*Fasting plasma glucose (mg/dl)*			
≤ 100	5197 (88.5%)	675 (11.5%)	0.001
100–126	1879 (88.5%)	386 (17.0%)	
> 126	1290 (76.3%)	401 (23.7%)	

◙CVDs include stroke, cardiac ischemic, and myocardial infarction. *Abnormal WC ≥ 102 cm (male) and ≥ 88 cm (female). **Normal WHR ≤ 0.85 (female) and ≤ 0.90 (male). ▀Normal HDL-C ≥ 40 mg/dl (male) and ≥ 50 mg/dl (women)

Chi-square test was used to examine the association between the variables and CVDs. *P*-value ≤ 0.05 was considered significant

The results showed a higher prevalence of inactivity than being active among participants with CVDs (20.7% vs. 10.7%, (*p* ≤ 0.001). The most prevalence of CVDs was found in participants with obesity (BMI > 30) (17.5%, *p* ≤ 0.001). In addition, there was a higher prevalence of abnormal compared to the normal WC and WHR (17.5 vs. 11%, *p* ≤ 0.001 and 15.6% vs. 8.2%, *p* ≤ 0.001, respectively) among participants with CVDs.

A lower prevalence of abnormal compared to normal cholesterol was shown among participants with CVDs (13.5% vs. 15.7%, *p* = 0.003). Conversely, a higher prevalence of abnormal compared to normal HDL was shown in CVDs (16.5% vs. 13.9%, *p* ≤ 0.001).

The prevalence of abnormal SBP (23.1% vs. 14.2%, *p* ≤ 0.001) and DBP (19.1% vs. 14.6%, *p* = 0.002) was more common in CVDs. The most prevalence of CVDs was among participants with FPG (> 126 mg/dl) compared to FPG (≤ 100 mg/dl) (23.7% vs. 11.5%, *p* = 0.001) compared to the normal FPG.

There were no significant differences between using a mobile (*p* = 0.243), drinking alcohol (*p* = 0.888), the levels of wealth index (*p* = 0.22) or TG levels in participants with CVDs (*p* = 0.521).

### Association of risk factors with CVD

The crude and adjusted analysis for evaluating the association between all characteristics and CVDs was presented in Table 3. The results of crude analyses were not presented in this part.

**Table 3:** Association between all characteristics of the study population and CVDs ◙

Characteristics	Crude		Adjusted ▼	
	OR (95% CI)	*P*-value	OR (95% CI)	*P*-value
Age (35- 45)				
45–55	2.18 (1.86, 2.54)	≤ 0.001	2.02 (1.72, 2.37)	≤ 0.001
55–65	3.93 (3.35, 4.59)	≤ 0.001	3.35 (2.82, 3.99)	≤ 0.001
65–70	4.79 (3.90, 5.87)	≤ 0.001	3.97 (3.14, 5.01)	≤ 0.001
Gender (Female)				
Male	1.37 (1.22, 1.54)	≤ 0.001	1.76 (1.51, 2.05)	≤ 0.001
Marital status (Single)				
Married	2.06 (1.37, 3.08)	≤ 0.001	1.63 (1.08, 2.47)	0.021
Widow	4.03 (2.61, 6.22)	≤ 0.001	1.57 (0.99, 2.41)	0.051
divorced	1.31 (0.68, 2.51)	0.418	0.88 (0.45, 1.71)	0.7
Wealth index (Poorest)				
Poor	0.98(0.82, 1.16)	0.787	0.87 (0.72,1.04)	0.113
Moderate	0.82 (0.69, 0.98)	0.031	1.03 (0.85,1.24)	0.78
Rich	0.91 (0.77, 1.09)	0.305	1.02 (0.84, 1.24)	0.86
Richest	0.96 (0.8, 1.14)	0.621	1.07 (0.88,1.29)	0.48
Using a mobile (No)				
Yes	0.92 (0.81, 1.05)	0.243	1.26 (1.09, 1.46)	0.002
Smoking cigarette (No)				
Yes	1.41 (1.24, 1.59)	≤ 0.001	1.44 (1.24, 1.68)	≤ 0.001
Drinking Alcohol (No)				
Yes	1.03 (0.69,1.52)	0.888	1.48 (0.97, 2.25)	0.067
Physical Activity Quantile 3 (Active)				
Quantile 1 (Inactive)	2.18 (1.9, 2.51)	≤ 0.001	1.56 (1.34, 1.8)	≤ 0.001
Quantile 2 (Moderate)	1.26 (1.09, 1.47)	0.002	1.1 (0.94, 1.28)	0.252
Body Mass Index (< 18.5) (kg/cm^2^)				
18.5–24.99	0.94 (0.57,1.55)	0.82	1.12 (0.67,1.87)	0.675
25–29.99	1.06 (0.65, 1.74)	0.8	1.3 (0.78,2.2)	0.314
> 30	1.41 (0.87, 2.31)	0.16	1.68 (1.01, 2.8)	0.047
Waist Circumference (WC) (cm) (Normal)				
Abnormal*	1.66 (1.46,1.88)	≤ 0.001	1.09 (0.89, 1.32)	0.414
Waist-to-Hip Ratio (WHR) (Normal) **				
Abnormal	2.06 (1.63, 2.61)	≤ 0.001	1.18 (0.91, 1.52)	0.213
Triglyceride ≥ 150 mg/dl				
< 150 mg/dl	0.96 (0.86,1.08)	0.521	0.88 (0.78, 1)	0.051
Cholesterol ≥ 200 mg/dl				
< 200 mg/dl	0.84 (0.74, 0.94)	0.003	0.69 (0.61, 0.79)	≤ 0.001
High-density lipoprotein cholesterol (HDL-C) (Abnormal)				
Normal▀	1.22 (1.09, 1.37)	0.001	1.1 (0.97,1.25)	0.583
Systolic Blood Pressure < 140 mmHg				
≥ 140 mmHg	1.82 (1.53, 2.17)	≤ 0.001	1.25 (1.04, 1.51)	0.017
Diastolic Blood Pressure < 90 mmHg				
≥ 90 mmHg	1.38 (1.12,1.7)	0.002	0.89 (0.78, 1.32)	0.89
Fasting Plasma Glucose ≤ 100 (mg/dl)				
100–126	1.58 (1.38, 1.81)	≤ 0.001	1.24 (1.07, 1.43)	0.003
> 126	2.39 (2.08, 2.75)	≤ 0.001	1.71 (1.47, 1.98)	≤ 0.001

CI = Confidence Interval; OR = Odds ratio

*P*-value < 0.05 was considered significant. ▼Adjusted for all variables in Table 3

◙Cardiovascular diseases include stroke, cardiac ischemic, and myocardial infarction. *Abnormal WC ≥ 102 cm (male) and ≥ 88 cm (female). **Normal WHR ≤ 0.85 (female) and ≤ 0.90 (male). ▀Normal HDL-C ≥ 40 mg/dl (male) and ≥ 50 mg/dl (women)

In the adjusted analyses, the participants aged 65–70 y showed the most odds of CVDs (OR: 3.97, 95% CI: (3.14, 5.01), (*p* ≤ 0.001)). The higher odds of CVDs were reported in the participants age 45–55 y (OR: 3.35, 95% CI: (2.82, 3.99), (*p* ≤ 0.001)) and 55–65 y (OR: 2.02, 95% CI: (1.72, 2.37), (*p* ≤ 0.001)) in comparison with the participants aged 35–45 y.

In addition, CVDs were associated with males (OR: 1.76, 95% CI: (1.51, 2.05), *p* ≤ 0.001), married status (OR: 1.63, 95% CI: (1.08, 2.47), *p* = 0.021), more using a mobile phone (OR: 1.26, 95% CI: (1.09, 1.46), *p* ≤ 0.002), and smoking cigarettes (OR: 1.44, 95% CI: (1.24, 1.68), *p* ≤ 0.001). Moreover, higher odds of CVDs were found in the participants with low PA (OR: 1.56, 95% CI: (1.34, 1.8), *p* ≤ 0.001) and BMI > 30 (kg/m^2^) (OR: 1.68, 95% CI: (1.01, 2.8), p ≤ 0.047). Further, SBP ≥ 140 mm Hg (OR: 1.25, 95% CI: (1.04, 1.51), *p* = 0.017), FPG = 100–126 mg/dl (OR: 1.24, 95% CI: (1.07, 1.43), *p* = 0.003), and FPG > 126 mg/dl (OR: 1.71, 95% CI: (1.47, 1. 98), *p* ≤ 0.001) were associated to CVDs. People with cholesterol < 200 mg/dl (OR: 0.69, 95% CI: (0.61, 0.79), *p* ≤ 0.001) showed lower odds of CVDs compared to whom with cholesterol > 200 mg/dl. It was found no odds of CVD with other factors, including wealth index, alcohol drinking, moderate PA, normal and overweight, WC, WHR, TG, HDL-C, DBP, widow, and divorced status.

## Discussion

The present findings showed: (1) the main risk factors of CVDs were age, married status, mobile phone use, low PA, smoking, obesity, and abnormal FPG and SBP; (2) lower odds of CVDs were shown in participants with normal cholesterol; (3) there was a high prevalence of the components of CVDs (CI, MI, and stroke), HTN, prediabetes, T2DM, overweight, and obesity in Hoveyzeh, especially among females; (4) The prevalence of CVDs was related to older age, female, widow, smoking cigarettes, low PA, DBP ≥ 90 mmHg, SBP ≥ 140 mmHg, abnormal WC, WHR, and FPG, and obesity.

In agreement with the present study, the previous studies reported the prevalence of CVDs was higher in Iranians with dyslipidemia [12]. Moreover, the prevalence of dyslipidemia, overweight, obesity, HTN, prediabetes, low PA, and current smoking was high in the Gulf region (Iran, Kuwait, Oman, United Arab Emirates, Bahrain, etc.) [13–15], which was similar the present findings. A meta-analysis (2018) conducted by Akbartabar Toori showed that dyslipidemia was high in the Iranian population of different races [12]. The present study showed the prevalence and risk factors of CVDs in the Arab community.

In the United Arab Emirates, a greater risk of CVDs was reported in men than women. According to the previous studies, the risk factors of CVDs include overweight, obesity, smoking, HTN, low PA, and DM in the United Arab Emirates and the Eastern Mediterranean region [12, 15, 16], which were similar to the result of the present study.

Higher odds of CVDs were related to an unhealthy lifestyle (smoking, mobile phone use, obesity, and low PA). However, the previous findings showed the positive effects of PA in reducing risk factors and the initiation of CVDs [16, 17], and details on the mechanism remain largely unclear.

The regular and appropriate PA and the type and amount of PA, regardless of gender and age, are the critical factors in CVDs that should be considered [18–20].

The protective effect of PA on CVDs might be mediated by improving metabolic and inflammatory risk markers and balance in energy expenditure, which finally leads to controlling HTN, diabetes, obesity, and reducing the risk of stroke, both ischemic and hemorrhagic [21, 22].

Apart from active smoking, passive smoking (exposure to environmental tobacco smoke) plays a significant role in CVDs [23]. Unfortunately, inactive smokers were not identified in the present research. Smoking cigarettes develop some risk factors for CVDs following as glucose intolerance, dyslipidemia, thrombus formation, inflammation, and oxidative stress as a potential mechanism for directly initiating CVDs [23, 24]. However, in the present study, higher odds of CVDs were related to BMI ≥ 30 kg/m^2^, the p-value was negligible (*p* = 0.047). The previous findings also showed obesity and, especially, severe obesity (BMI ≥ 35 kg/m^2^) were consistently and strongly related to a higher risk of CVDs [25]; still, the duration of obesity or delaying the onset of obesity may have significant CV health benefits. Therefore, it should not be assumed that only obesity is the leading risk factor for CVDs regardless of other metabolic profiles.

A meta-analysis showed overall correlations between CVD risk factors and anthropometric adiposity measures, including BMI, WC, WHR, waist-to-height ratio, and body fat percentage, had little significance. Moreover, the measurement of WC was more related to CVD risk factors in men and women than BMI [24] in disagrees with the present results.

However, the previous studies showed that increased TG [25] and low HDL-C were associated with an increased risk of CVDs, [25, 26]; TG and HDL-C did not have any significant changes related to CVDs risk in the present study. Moreover, the higher prevalence of CVDs was not shown in participants with abnormal TG. It may be due to taking medications from the participants with CVDs.

Therefore, it seems necessary to evaluate other lipid profiles such as total cholesterol/HDL-C and TG/HDL-C ratio and Apolipoproteins [25].

Insulin resistance is a major feature of T2DM. Moreover, it is associated with several metabolic abnormalities such as elevated CVDs risk. Therefore, independent of glucose control, improving insulin sensitivity is expected to reduce dysglycemia, which is considered the risk of CVDs in patients with T2DM [27].

### Limitations and strengths

The present study had some limitations, including (1) the age of the participants was restricted to the 35–70-year age group; therefore, the results cannot be generalized to younger or older age groups; (2) the estimated prevalence of CVDs may be reported less than reality due to only CI, MI, and stroke were considered as CVDs (other types of CVDs include coronary heart disease, peripheral arterial disease, rheumatic heart disease, congenital heart disease, etc. were not evaluated); (3) As it was a cross-sectional study, the causal relationships could not have been established between the risk factors and CVDs; (4) sociodemographic information may induce measurement error due to self-reported collection. However, it is noteworthy that it was tried to minimize the errors with strict quality control of data collection procedures by extensively the trained collectors (5) the evaluation of other lipid profiles (total cholesterol/HDL-C and TG/HDL-C ratio and Apolipoproteins) may be to more accurately estimate CVD risk factors [25]

The strengths of the present studies were the large sample size, which includes both males and females, and a wide range of variables, including demographic, socioeconomic, lifestyle, clinical, and biochemical factors likely to be associated with CVDs was collected from the participants. In addition, it was the first time a large sample size from the Arab community of Iran was evaluated. Moreover, the participants were from Iran, a country in the Gulf area, the Middle East, where the publication of CVD papers still lags behind developed countries and needs to conduct more excellent research [28].

It is no secret that there are other risk factors such as dietary intake [25], resting heart rate [29], other biochemical parameters (insulin resistance [27], LDL, total cholesterol/HDL-C and TG/HDL-C ratio and Apolipoproteins [25]) that should be evaluated in this population. Furthermore, the present findings can have generalized to a large area of southwestern Iran and southern Iraq due to having similarities in terms of race, ethnicity, culture, and lifestyle. Furthermore, the findings controlled multiple potential confounders using multivariable logistic regression models. Finally, our results provided valuable information for the health policymakers to prevent and control CVDs by identifying the risk factors for CVDs.

Given that some risk factors were controllable and preventable; health policymakers should pay further attention to amending them to reduce the prevalence of CVDs in Iran. Moreover, the role of gender in the incidence of CVDs is notable due to the function of various genes which regulate telomerase activity and sexual hormones (estrogens) [17].

## Conclusion

Among Hoveyzeh adults, the greater odds of CVDs were related to older age, married status, mobile phone use, low PA, smoking, obesity, and abnormal FPG and SBP. Moreover, lower odds of CVDs were related to normal cholesterol; 3) The prevalence of CVDs was related to older age, female, widow, smoking cigarettes, low PA, DBP ≥ 90 mmHg, SBP ≥ 140 mmHg, abnormal WC, WHR, and FPG, and obesity. The present findings showed that the preventable risk factors of CVDs, including unhealthy lifestyles, play a sustainable role. Hence, the health ministry's agenda should be to design and implement long-term public health programs to achieve a healthy lifestyle. It also suggests that similar studies will be conducted in neighborhood countries on the Arab community to identify the role of race, ethnicity, and culture.

## Supplementary Information

**Additional file1: Table S1: **General questionnaire including demographic, socioeconomic status, behavior habits, and medical history.

## Data Availability

The datasets used and/or analyzed during the current study are available from the corresponding author on reasonable request.

## Abbreviations

BMI: Body mass index
CI: Cardiac ischemic
CVDs: Cardiovascular diseases
DBP: Diastolic blood pressure
DALYs: Disability-adjusted life years
FPG: Fasting plasma glucose
HC: Hip circumferences
HCS: Hoveyzeh Cohort Study
HDL-C: High-density lipoprotein cholesterol
MI: Myocardial infarction
PA: Physical activity
PERSIAN: Prospective epidemiological studies of the Iranian Adult and Neonates
SBP: Systolic blood pressure
SD: Standard deviations
T2DM: Type 2 diabetes mellitus
TC: Total cholesterol
TG: Triglyceride
WC: Waist circumference
WHO: World health organization
WHR: High waist-to-hip ratio
