# Supplementary material for: Prevalence of cardiovascular diseases and associated factors among adults from southwest Iran: Baseline data from Hoveyzeh Cohort Study
Source: BMC Cardiovasc Disord. 2022 Jul 8;22:309. doi: 10.1186/s12872-022-02746-y (PMC9270737; doi:10.1186/s12872-022-02746-y)
Supplement: Supplementary file 1 — Additional file1: Table S1: General questionnaire including demographic, socioeconomic status, behavior habits, and medical history. [file 12872_2022_2746_MOESM1_ESM.docx]

**Table S.1:** General questionnaire including demographic, socioeconomic status, behavior habits, and medical history

| **Name:** | | | | **Family name:** | | | | **PCID:** | | | |
| --- | --- | --- | --- | --- | --- | --- | --- | --- | --- | --- | --- |
| **Age:**…..years | **Wealth Score:** poorest□ poor□ moderate□ rich□ richest □ | | | | | | | | | **Gender**: Female□ Male□ | |
| **Material status:** Married □ Single□ Widow□ Divorced □ | | | | | | | | | **Using mobile** Yes No | | |
| **Weight:** … Kg | | **Height:** …..cm | | | **Body mass index (BMI):** …. | | **Hip Circumference:** ….Cm | | | | |
| **Waist circumstance:**...Cm | | | **Waist-to-hip ratio:**….Cm | | | **DBP:** ….mmHg | | | | | **SBP**: ….mmHg |
| **Smoking habits** | | | | | | | |  | | | |
| Do you smoke more than 100 cigarettes? | | | | | | | | Yes No | | | |
| How old were you when you first started smoking? | | | | | | | | … years | | | |
| How old were you when you first started smoking? | | | | | | | | … years | | | |
| Do you smoke now? | | | | | | | | Yes No | | | |
| On average, how many cigarette per day do you smoke? | | | | | | | | ……cigarette | | | |
| At what age did you stop smoking? | | | | | | | | … years | | | |
| Did anyone in your family smoke during your childhood? | | | | | | | | Yes No | | | |
| Have you ever been exposed to cigarette smoke at home or are you? | | | | | | | | Yes I don’t know Almost Never | | | |
| How many hours per day are you exposed to cigarette smoke at home? | | | | | | | | …..hours | | | |
| Have you ever been exposed to cigarette smoke at work? | | | | | | | | Yes I don’t know Almost Never | | | |
| How many hours per day are you exposed to cigarette smoke at work? | | | | | | | | …..hours | | | |
| Have you ever used chibouk, pipe Tabaco, nas, shisha? | | | | | | | | Yes I don’t know Almost Never | | | |
| Have you ever been used drugs? | | | | | | | | Yes No | | | |
| Have you ever been drank alcohol? | | | | | | | | Yes No | | | |
| **Sleep habits** | | | | | | | |  | | | |
| What time have you gone to bed at night? | | | | | | | |  | | | |
| How long has it usually take to fall asleep each night? | | | | | | | | …..min | | | |
| What time have you usually gotten up in the morning? | | | | | | | |  | | | |
| How long has it usually take to get up out of the bed in the morning | | | | | | | | …..min | | | |
| What time do you like to get up in the morning? | | | | | | | |  | | | |
| How long do you like to take to get up out of the bed in the morning | | | | | | | | …..min | | | |
| Do you usually take naps during the day? | | | | | | | | Yes No | | | |
| How often do you nap during the day? | | | | | | | | …….min | | | |
| Do you have Night Working Shift? | | | | | | | | Yes No | | | |
| How many times a year do you have night shifts? | | | | | | | | ……times | | | |
| Do you have Leg Restlessness At Sleep? | | | | | | | | Yes No | | | |
| Do you Unintentionally nap during the day? | | | | | | | | Yes No | | | |
| Have you used Sleeping Pills? | | | | | | | | Yes No | | | |
| How long does your sleep duration? | | | | | | | | ……hours | | | |
| **History of diseases** | | | | | | | |  | | | |
| History of Cardiac Ischemic | | | | | | | |  | | | |
| How old were you when Cardiac Ischemic Started? | | | | | | | | Yes No | | | |
| Are you under treatment of Cardiac Ischemic? | | | | | | | | Yes No | | | |
| History of [Myocardial infarction](https://en.wikipedia.org/wiki/Myocardial_infarction) | | | | | | | | Yes No | | | |
| How old were you when [Myocardial infarction](https://en.wikipedia.org/wiki/Myocardial_infarction) Started? | | | | | | | | …..years | | | |
| Are you under treatment of [Myocardial infarction](https://en.wikipedia.org/wiki/Myocardial_infarction)? | | | | | | | | Yes No | | | |
| History of Stroke | | | | | | | | Yes No | | | |
| How old were you when Stroke Started? | | | | | | | | …..years | | | |
| Are you under treatment of Stroke? | | | | | | | |  | | | |
| **Physical activity** | | | | | | | |  | | | |
| How many activity patterns do you use during the year? | | | | | | | | N=…. | | | |
| How much is your average night's sleep during the 24 hours? | | | | | | | | …..: ….: ……. | | | |
| During the 24 hours, how much time do you sleep in the evening or on the days? | | | | | | | | …..: ….: ……. | | | |
| During the 24 hours, how much time do you spend watching TV, listening to music, watching movies on the computer and or the like? | | | | | | | | …..: ….: ……. | | | |
| During the 24 hours, how much time do you spend reading the books, magazines, newspapers or the like, are you? | | | | | | | | …..: ….: ……. | | | |
| During the 24 hours, how much time do you spend doing office work sitting at your desk? | | | | | | | | …..: ….: ……. | | | |
| During the 24 hours, how much time do you spend working with the computer (behind the desk or in any other situation)? | | | | | | | | …..: ….: ……. | | | |
| During the 24 hours, how much times | | | | | | | | …..: ….: ……. | | | |
| During the 24 hours, how much time do you spend eating, sitting in meetings, parties, conferences or the like? | | | | | | | | …..: ….: ……. | | | |
| During the 24 hours, how much time do you spend cooking, washing dishes, standing and the like? | | | | | | | | …..: ….: ……. | | | |
| During the 24 hours, how much time do you spend driving (motor vehicle, sitting, operator work) or doing the similar driving activities? | | | | | | | | …..: ….: ……. | | | |
| During the 24 hours, how much time how long do you spend Light housekeeping? | | | | | | | | …..: ….: ……. | | | |
| During the 24 hours, how much time walking, walking slowly, going down or up stair, exercise, rhythmic activities or the like? | | | | | | | | …..: ….: ……. | | | |
| During the 24 hours, how much times do you spend walking fast, light aerobics, recreational cycling, cycling to get from home to work, or the like? | | | | | | | | …..: ….: ……. | | | |
| During the 24 hours, how much time do you spend doing things like driving agricultural implements, road construction machinery, or any similar activity? | | | | | | | | …..: ….: ……. | | | |
| During the 24 hours, how much time do you spend in light technical jobs (oil change, car service, car wash, car paint services, battery making, etc.)? | | | | | | | | …..: ….: ……. | | | |
| During the 24 hours, how much time do you spend moving furniture, carrying light objects over bridges, or any similar activity? | | | | | | | | …..: ….: ……. | | | |
| During the 24 hours, how much time do you spend gardening, light farming activities, or any similar activity? | | | | | | | | …..: ….: ……. | | | |
| During the 24 hours, how much time do you spend doing jobs such as heavy technical jobs (car engine services, blacksmithing, turning, casting, etc.), working in sawmills or any similar activity? | | | | | | | | …..: ….: ……. | | | |
| During the 24 hours, how much time do you spend doing heavy labor or agricultural activities (Snow shoveling, carrying heavy objects from bridges) | | | | | | | | …..: ….: ……. | | | |
| During the 24 hours, how much time do you spend doing exercises such as bodybuilding, strenuous aerobics, or any similar activity? | | | | | | | | …..: ….: ……. | | | |
